# On testing structural identifiability by a simple scaling method: Relying on scaling symmetries can be misleading

**DOI:** 10.1371/journal.pcbi.1009032

**Published:** 2021-10-14

**Authors:** Alejandro F. Villaverde, Gemma Massonis

**Affiliations:** 1 Universidade de Vigo, Department of Systems Engineering and Control, Vigo, Galicia, Spain; 2 Universidade de Vigo, Department of Applied Mathematics II, Vigo, Galicia, Spain; 3 Bioprocess Engineering Group, IIM-CSIC, Vigo, Galicia, Spain; Inria, FRANCE

## Abstract

A recent paper published in *PLOS Computational Biology* [1] introduces the Scaling Invariance Method (SIM) for analysing structural local identifiability and observability. These two properties define mathematically the possibility of determining the values of the parameters (identifiability) and states (observability) of a dynamic model by observing its output. In this note we warn that SIM considers scaling symmetries as the only possible cause of non-identifiability and non-observability. We show that other types of symmetries can cause the same problems without being detected by SIM, and that in those cases the method may lead one to conclude that the model is identifiable and observable when it is actually not.

## Notation and definitions

Consider an ordinary differential equation (ODE) model given by:

$$\begin{array}{c} M:=\{\begin{array}{cc} \dot{x}\left(t\right) & =f(x(t),\lambda ,u(t))\;,\\ y(t) & =h(x(t),\lambda ,u(t))\;. \end{array} \end{array}$$

where *x*(*t*), λ, *u*(*t*), and *y*(*t*) are the state, parameter, input, and output vectors, respectively, and *f* and *h* are analytic vector functions. Assuming perfect knowledge of *u*(*t*) and *y*(*t*), the *i*^th^ component λ_*i*_ of the parameter vector λ is *structurally locally identifiable* if for almost any λ* there is a neighbourhood $N(\lambda^{*})$ and an admissible input *u*(*t*) for which the following property holds:

$$\hat{\lambda }\in N\left(\lambda^{*}\right)\;\text{and}\;y\left(t,\hat{\lambda }\right)=y\left(t,\lambda^{*}\right)\Rightarrow {\hat{\lambda }}_{i}=\lambda_{i}^{*}\;.$$

Similarly, a state *x*_*i*_ is *observable* if its initial value *x*_*i*_(*t*_0_) can be determined from *y*(*t*) and *u*(*t*) in some finite time *t*_*f*_ > *t*_0_.

A parameter is structurally *locally* identifiable if its value can be distinguished from other values in its neighbourhood, but not necessarily from other discrete transformations. In contrast, structurally *globally* identifiable parameters are uniquely determined in the whole parameter space. The SIM test analysed in this paper is in the category of methods that study structural local identifiability.

It should be noted that structural local identifiability and observability are properties that hold for *almost all* points in parameter or state space—that is, with the possible exception of a subset of measure zero. Likewise, these properties may not hold for all the possible input vectors—the above definitions entail that at least a subset of the admissible inputs is sufficiently exciting for that purpose.

## Description of the SIM test

The existence of symmetries in the equations of a dynamic model is one reason a model may not be structurally identifiable and observable. Such symmetries [2, 3] allow for similarity transformations [4], that is, transformations of parameters and state variables that leave the model output invariant [5]. Since the parameters and states involved in such symmetries cannot be distinguished by observing the output, they are unidentifiable and unobservable, respectively.

The SIM test proposed in [1] begins by decomposing the dynamic equations of a model as a sum of functionally independent functions. That is, the ordinary differential equation of each state is expressed as:

$$\begin{array}{c} {\dot{x}}_{i}=\sum_{k=1}^{M}f_{ik}\left({\tilde{x}}_{k},{\tilde{\lambda }}_{k}\right) \end{array}$$

where ${\tilde{x}}_{k},{\tilde{\lambda }}_{k}$ are the subsets of states and parameters that appear in *f*_*ik*_, and each *f*_*ik*_ is functionally independent of *f*_*il*_, meaning that they satisfy the generalized Wronskian theorem [6]. SIM assumes that the model output consists of a subset of the state variables, i.e. the observation function must be of the form *y*(*t*) = *h*(*x*(*t*)) = *x*_*Y*_(*t*), where *x*_*Y*_(*t*) is a subset of the elements of *x*(*t*). Next, unknown parameters λ_*i*_ and unobserved states *x*_*j*_ are multiplied by unknown scaling factors,

$$\begin{array}{c} \lambda_{i}\to u_{\lambda_{i}}\lambda_{i},\\ x_{j}\to u_{x_{j}}x_{j} \end{array}$$

and each functionally independent function is equated to its scaled version,

$$\begin{array}{c} f_{ik}\left(\tilde{x},\tilde{\lambda }\right)=\frac{1}{u_{x_{i}}}f_{ik}\left(u_{\tilde{x}}\tilde{x},u_{\tilde{\lambda }}\tilde{\lambda }\right) \end{array}$$

We remark that the observed—i.e. directly measured—states must not be multiplied by scaling factors. Finally, combinations of the scaling factors that leave the equations invariant are sought. The SIM classifies the parameters (respectively, state variables) for which the above equations imply $u_{\lambda_{i}}=1$ as structurally locally identifiable (respectively, observable, $u_{x_{i}}=1$).

Thus, the SIM approach to analysing structural identifiability and observability is to search for a particular type of symmetry, namely scaling symmetries. If a model only contains scaling symmetries, or if it does not contain symmetries of any kind, SIM provides a correct result. However, other types of symmetries can also be present in ordinary differential equation models. A number of examples from biology have been discussed in the recent literature, see e.g. [7–9]. If the equations of a model only have symmetries that are not of the scaling type, the SIM test does not detect them and wrongly classifies the related parameters as structurally identifiable and the related state variables as observable.

SIM’s limitation to scaling symmetries is mentioned in [1] (“our identifiability test (…) provides a simple way to find a type of symmetry that is related to scale invariance”). However, that paper does not mention that due to this limitation the SIM test can yield wrong results; instead, it claims that “scaling invariance of the model equations can be used to determine whether the parameters are unidentifiable or not”. Indeed, the existence of a scaling invariance indicates that the parameters are unidentifiable. However, the opposite is not true, i.e. its absence does not mean that the parameters are identifiable. We discuss two counter-examples to illustrate this risk.

## Counter-example 1: The FitzHugh-Nagumo model

We first consider the classical FitzHugh-Nagumo model, whose structural identifiability and symmetries were discussed in [7]. It is a nonlinear model that can describe an excitable system such as a neuron, and it can exhibit oscillatory behaviour. Its equations are:

$${\dot{x}}_{1}\left(t\right)=c(x_{1}\left(t\right)-\frac{x_{1}^{3}\left(t\right)}{3}-x_{2}\left(t\right)+d),$$ (1)

$${\dot{x}}_{2}\left(t\right)=\frac{1}{c}(x_{1}\left(t\right)+a-b\cdot x_{2}\left(t\right)),$$ (2)

$$y(t)=x_{1}\left(t\right)$$ (3)

where the *x*_*i*_(*t*) are the states, *y*(*t*) is the measurable output, and *a*, *b*, *c*, *d* are unknown parameters. The initial condition of the first state, *x*_1_(*t* = 0), is known, because it is a directly measured state; *x*_2_(*t* = 0) is unknown. In what follows we omit the dependency on *t* to simplify the notation.

We show the calculations of the SIM test below. Briefly, each functionally independent term in (1) and (2) is equated to its scaled counterpart, which introduces scaling factors u_*_ for every state and parameter (except for *x*_1_, which is directly measured). The scaled terms in the ODE of ${\dot{x}}_{2}$ are divided by $u_{x_{2}}$ to account for the fact that the derivative of the state is also scaled. The procedure yields the following equations, where (4)–(7) come from (1) and (8)–(10) come from (2):

$$u_{c}\cdot c\cdot x_{1}=c\cdot x_{1}\Rightarrow u_{c}=1,$$ (4)

$$-u_{c}\cdot c\cdot \frac{x_{1}^{3}}{3}=-c\cdot \frac{x_{1}^{3}}{3},$$ (5)

$$u_{c}\cdot c\cdot u_{x_{2}}\cdot x_{2}=c\cdot x_{2}\Rightarrow u_{x_{2}}=1,$$ (6)

$$u_{c}\cdot c\cdot u_{d}\cdot d=c\cdot d\Rightarrow u_{d}=1,$$ (7)

$$\frac{x_{1}}{cu_{c}u_{x_{2}}}=\frac{x_{1}}{c},$$ (8)

$$\frac{1}{u_{x_{2}}}\frac{u_{a}\cdot a}{u_{c}\cdot c}=\frac{a}{c}\Rightarrow u_{a}=1,$$ (9)

$$\frac{1}{u_{x_{2}}}\frac{u_{b}\cdot b\cdot u_{x_{2}}\cdot x_{2}}{u_{c}\cdot c}=\frac{b\cdot x_{2}}{c}\Rightarrow u_{b}=1$$ (10)

The implications in the above equations are deduced in cascade, i.e. the finding from (4) that *u*_*c*_ = 1 is used in (6) to obtain $u_{x_{2}}=1$, and so on. Note that these results are valid for almost all values of the involved parameters and states, but not if they are zero. Under these conditions, since the only possible solution is the trivial one (i.e. all the scaling factors are equal to one), the SIM test classifies this model as structurally locally identifiable (s.l.i.) and observable.

However, this result is incorrect: the model is in fact unidentifiable and unobservable, due to the existence of an affine symmetry [7]. This result, which we obtained with the STRIKE-GOLDD toolbox [10], can be easily verified analytically, as we now show. The symmetry analysis, performed using the procedure described in [9], finds the following symmetries, expressed as one-parameter Lie groups of transformations:

$$x_{2}^{*}⟶x_{2}+\varepsilon$$ (11)

$$a^{*}⟶a+b\cdot \varepsilon$$ (12)

$$d^{*}⟶d+\varepsilon$$ (13)

The symmetries are defined as a function of a new parameter *ε*. The above expressions (11)–(13) indicate that replacing the terms to the left of the arrow with their right hand equivalents does not modify the model output. Replacing the right hand expressions of (11)–(13) in the model ODEs (1) and (2) it is immediate to see that the above transformations leave the model equations invariant:

x˙1=c(x1−x133−x2−ε+d+ε), (14)

x˙2=1c(x1+a+b·ε−b(x2+ε)) (15)

Thus, the state *x*_2_ and the parameters *a*, *d* cannot be distinguished from their transformed values (11)–(13), and are therefore unobservable and structurally unidentifiable, respectively. However, since the cause of this lack of structural identifiability and observability is not a scaling symmetry but an affine one, SIM wrongly classifies them as observable and structurally identifiable.

## Counter-example 2: A linear compartment model

This second case study shows that not only nonlinear models can have non-scaling symmetries. The following linear compartment model was presented as Example 6.3 in [11], where it was reported that it is unidentifiable and has no identifiable scaling reparameterization. The model consists of four states (one of which is directly measured, *x*_1_), ten parameters, and one known input, *g*, which is a generic analytic function. As in the previous example, only the initial condition of the measured state, *x*_1_(*t* = 0), is known. The initial conditions of the other states are assumed to have generic unknown values.

$${\dot{x}}_{1}=a_{11}\cdot x_{1}+a_{12}\cdot x_{2}+g,$$ (16)

$${\dot{x}}_{2}=a_{21}\cdot x_{1}+a_{22}\cdot x_{2}+a_{23}\cdot x_{3},$$ (17)

$${\dot{x}}_{3}=a_{33}\cdot x_{3}+a_{34}\cdot x_{4},$$ (18)

$${\dot{x}}_{4}=a_{42}\cdot x_{2}+a_{43}\cdot x_{3}+a_{44}\cdot x_{4},$$ (19)

$$y=x_{1}$$ (20)

The model above is structurally unidentifiable and non-observable due to the existence of scaling symmetries and one higher order Lie symmetry. Reformulating the model by removing the scaling symmetries, it is possible to obtain an equivalent model with only seven parameters:

$${\dot{x}}_{1}=a_{11}\cdot x_{1}+x_{2}+g,$$ (21)

$${\dot{x}}_{2}=a_{21}\cdot x_{1}+a_{22}\cdot x_{2}+x_{3},$$ (22)

$${\dot{x}}_{3}=a_{33}\cdot x_{3}+a_{34}\cdot x_{4},$$ (23)

$${\dot{x}}_{4}=x_{2}+a_{43}\cdot x_{3}+a_{44}\cdot x_{4}$$ (24)

where the measured output is again *y* = *x*_1_. By applying the SIM procedure we obtain the following equations:

$$x_{1}a_{11}u_{a_{11}}=x_{1}a_{11}\Rightarrow u_{a_{11}}=1,$$ (25)

$$x_{2}u_{x_{2}}=x_{2}\Rightarrow u_{x_{2}}=1,$$ (26)

$$x_{1}u_{a_{21}}a_{21}/u_{x_{2}}=a_{21}x_{1},$$ (27)

$$u_{a_{22}}a_{22}x_{2}=a_{22}x_{2}\Rightarrow u_{a_{22}}=1,$$ (28)

$$u_{x_{3}}/u_{x_{2}}x_{3}=x_{3},$$ (29)

$$u_{a_{33}}a_{33}x_{3}=a_{33}x_{3}\Rightarrow u_{a_{33}}=1,$$ (30)

$$u_{a_{34}}a_{34}x_{4}u_{x_{4}}/u_{x_{3}}=x_{4}a_{34},$$ (31)

$$u_{x_{2}}x_{2}/u_{x_{4}}=x_{2},$$ (32)

$$u_{a_{43}}a_{43}u_{x_{3}}x_{3}/u_{x_{4}}=a_{43}x_{3},$$ (33)

$$u_{a_{44}}a_{44}x_{4}=a_{44}x_{4}\Rightarrow u_{a_{44}}=1.$$ (34)

Note that, since *x*_1_ is measured and *g* is a known input, no scaling factors are introduced for them. Replacing the values obtained in (25), (26), (28), (30), and (34) in the remaining equations yields:

$$u_{a_{21}}/u_{x_{2}}=1\overset{\left(26\right)}{\Rightarrow }u_{a_{21}}=1,$$ (35)

$$u_{x_{3}}/u_{x_{2}}=1\overset{\left(26\right)}{\Rightarrow }u_{x_{3}}=1,$$ (36)

$$u_{x_{3}}/u_{x_{4}}=1\overset{\left(36\right)}{\Rightarrow }u_{x_{4}}=1,$$ (37)

$$u_{a_{34}}u_{x_{4}}/u_{x_{3}}=1\overset{\left(36,37\right)}{\Rightarrow }u_{a_{34}}=1,$$ (38)

$$u_{a_{43}}u_{x_{3}}/u_{x_{4}}=1\overset{\left(36,37\right)}{\Rightarrow }u_{a_{43}}=1.$$ (39)

where the numbers above the ⇒ operator indicate the equations used in the derivations. Thus, we obtain that all $u_{x_{j}}=u_{\lambda_{i}}=1$ and, consequently, the SIM test classifies this model as structurally locally identifiable and observable.

However, this result is incorrect: in fact, only *a*_11_, *a*_21_, *a*_22_ and *a*_34_ are s.l.i.. The remaining parameters (*a*_33_, *a*_43_ and *a*_44_) are structurally unidentifiable, and *x*_4_ is non-observable. As in the previous example, we can verify the correctness of this result (obtained with the STRIKE-GOLDD toolbox) by examining the symmetries of the model, which can be written as follows:

$$x_{4}^{*}⟶x_{3}\cdot \varepsilon +x_{4}$$ (40)

$$a_{33}^{*}⟶-a_{34}\cdot \varepsilon +a_{33}$$ (41)

$$a_{43}^{*}⟶\varepsilon \left(a_{33}-a_{44}\right)-a_{34}\cdot \varepsilon^{2}+a_{43}$$ (42)

$$a_{44}^{*}⟶a_{34}\cdot \varepsilon +a_{44}$$ (43)

Following the same procedure as in the previous example, it can be checked that, by replacing in the model Eqs (23) and (24) the left hand side of (40)–(43) with the right hand side, the transformations are cancelled and we obtain the original equations again (calculations not shown). This proves that the model is structurally unidentifiable, and therefore the result of the SIM test is incorrect.

## Simple methods are appealing, but their applicability must be examined with caution

The previous example demonstrates that even relatively simple models can have symmetries that make them unidentifiable and non-observable, and which are not of the scaling type. The counter-examples have also shown that, while the SIM test does not find these symmetries, it is possible to analyse the structural identifiability and observability of these models with symbolic computation methods. These results prompt us to comment on another aspect of [1], namely its assessment of the performance of computational methods. That paper analyses thirteen models with a number of symbolic computation methods, which, when compared to SIM, are portrayed as less applicable, less conclusive, and/or producing incompatible results. However, we have found that the performance of at least some of those computational tools is misrepresented. Whereas a fair comparison of computational times is generally hard to establish due to dependence on computational platforms and implementation/tuning details, correctness of the results can be established more objectively. In particular, we have examined the case of the STRIKE-GOLDD toolbox; other available computational tools include COMBOS [12], DAISY [13], EAR [14], GenSSI [15], ObservabilityTest [16], and SIAN [17]. In [1] it is claimed that the STRIKE-GOLDD toolbox cannot analyse four of the models and yields wrong results for a fifth one. In contrast, we analysed these five models with STRIKE-GOLDD and obtained conclusive and correct results in all cases. The files that reproduce our calculations can be downloaded from https://www.biorxiv.org/content/10.1101/2020.11.30.403956v1.supplementary-material; implementations of the two case studies analysed in this paper are also provided in the link. The issues reported in [1] suggest an incorrect use of the toolbox; however, without additional information we prefer to leave the speculations about their specific causes to the reader.

The ability to perform calculations by hand is a desirable feature, not only due to the convenience of not requiring a computing environment, but also because this process can provide unique insights about a problem. In this regard, the SIM test proposed in [1] is appealing and, indeed, it yields correct results in many cases. Unfortunately, it also gives wrong results in other cases, without providing any hint whatsoever. As this note has shown, even apparently simple models can have non-scaling symmetries for which SIM fails. Therefore, SIM can be used as a preliminary test for structural *unidentifiability*. If the test classifies a model as identifiable, the result is inconclusive, and it should be double-checked with a different method.

Structural identifiability and observability are properties that often defy intuition, and the search for a simple approach to analyse them has proven elusive for decades. Their analysis usually entails complex symbolic computations that require specialized software. Fortunately, there is a number of well-established tools, available in a variety of computing environments, which can help in this endeavour.
